# Serial blood volume estimation in rabbits using trivalent chromium – An exploratory study

**DOI:** 10.1016/j.mex.2019.05.009

**Published:** 2019-05-10

**Authors:** Prathap Moothamadathil Baby, Pramod Kumar, Rajesh Kumar, Sanu Susan Jacob, Dinesh Rawat, Binu VS, Kalesh M. Karun, Nishanth B. Bhat

**Affiliations:** a Department of Physiology, Melaka Manipal Medical College (Manipal Campus), Manipal Academy of Higher Education, Karnataka, India; b Department of Plastic Surgery, Kasturba Medical College – Manipal, Manipal Academy of Higher Education, Karnataka, India; c Department of Radiotherapy Oncology, Kasturba Medical College – Manipal, Manipal Academy of Higher Education, Karnataka, India; d Department of Physiology, Kasturba Medical College – Manipal, Manipal Academy of Higher Education, Karnataka, India; e Department of Statistics, Manipal Academy of Higher Education, Manipal, Karnataka, India; f Department of Microbiology, Melaka Manipal Medical College (Manipal Campus), Manipal Academy of Higher Education, Karnataka, India

**Keywords:** Technique for repeat blood volume measurement, Blood volume, Trivalent chromium, New Zealand white rabbits, Repeat blood volume, Capillary leakage, Volume shift, Sepsis

## Abstract

Though often used in cardiac intensive monitoring set up, simultaneous evaluation of several variables like CVP, PCWP, SVV and hTEE for fluid volume resuscitation (especially when capillary permeability is major problem than cardiac performance) is a major challenge in many ICU setups. Therefore, repetitive determination of blood volume by trivalent chromium [^51^Cr (III)] as a direct single variable method may be a near ideal method during fluid volume resuscitation in cases where capillary permeability is a major problem (e.g. Burns). Hence, in the present article the repeatability and reliability of ^51^Cr (III) method in New Zealand white rabbits was explored. Mean blood volume values of initial measurement were 195.66 ± 47.30 ml or 89.81 ± 17.88 ml/kg body weight. Repeated mean blood volume values, measured after 1 h, was 181.98 ± 53.16 ml or 83.68 ± 22.09 ml/kg body weight. The average difference between the initial and repeated measurements was 10.93 ml (95% CI −3.33, 25.19 ml), which is not statistically significant (P = 0.128). The method using ^51^Cr (III) for repeat blood volume measurements after sixty minutes in rabbits is a reliable method.

• Rapid
• Repeatable
• Reliable

## Graphical abstract

**Figure:**
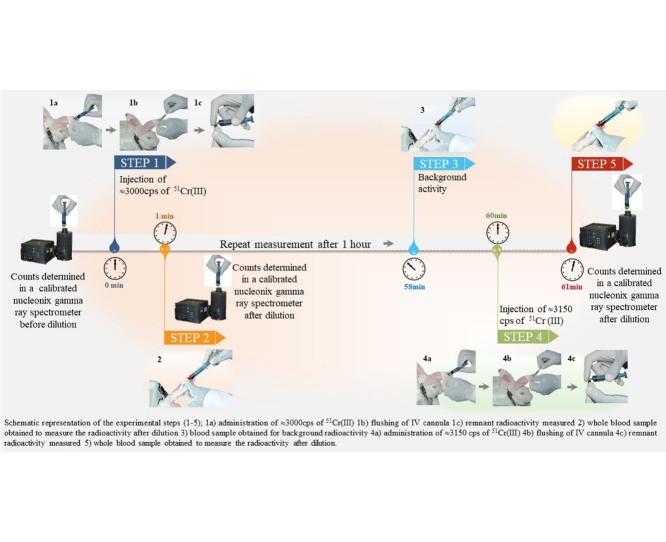

**Specifications Table**

**Table:** 

Subject Area:	*Medicine and Dentistry*
More specific subject area:	*Physiology* *Plastic Surgery*
Method name:	*Technique for repeat blood volume measurement*
Name and reference of original method:	*Blood volume measurement using trivalent chromium* *Indian J Plast Surg. 2014 May;47(2):242-8.*
Resource availability:	*The data sets used and/ or analysed during the current study are available from the corresponding author on reasonable request*

## Background

Capillary permeability with or without diminished cardiac function is the basic pathophysiological change in extensive burn patients and in sepsis. This poses a challenge to burn surgeons during monitoring and resuscitation. Currently used central venous pressure (CVP) and pulmonary capillary wedge pressure (PCWP) for volume status are unreliable to detect hypovolemia or hypervolemia [1,2]. In such instances, BV measurement remains useful as a clinical indicator in critically ill patients with extensive burns, profuse diarrhoea and in cases of high blood loss surgeries. In burn patient monitoring during fluid resuscitation, often CVP and PCWP is contraindicated and not possible. Also, performing and analysis of SVV and hTEE may be a challenge in the burn ICU patients with extensive wound.

In the last 5 years, there exists no reports available for repetitive measurements of BV. The major hurdles associated with repetitive BV measurements include increased accumulation of radioactive tracer and inaccurate results due to faster clearance [3]. Thus, lack of repetitive measurement related BV studies coupled with dependence on inaccurate CVP and PCWP based measurements, has been a major challenge in prompt management of patient with BV imbalance. Hence, there is a need to develop instant repeatable direct blood volume estimation by reliable dilution method where we can inject the desired material intravenously and collect the sample within minutes for determining dilution and calculating the blood volume.

## Materials and methods

All the animal experimental protocol were approved by the institutional animal ethics committee (IAEC/KMC/07/2007–2008). New Zealand white rabbits (Oryctolagus cuniculus), (n = 31; male n = 10, female n = 21), over two months of age with average weight of 2.21 kg were used for the study. All rabbits were provided with 12:12 h light-dark cycle, with 28 ℃ temperature and relative humidity between 30–70% in the animal housing. Two rabbits of the same sex were hygienically housed in a stainless steel cage and the collection pans were cleaned daily with water. Rabbits were fed with food and water adlibitum, except during the experiment.

The detailed methodology for BV measurement using trivalent chromium and cannulation of rabbit ear has been described in earlier reports [4,5]. Two intravenous (IV) cannulae, of size 24 and 26 GA (BD Neoflon TM Singapore) were inserted, one on each ear, for drug injection and blood collection. 2 mCi of ^51^Cr in aqueous solution (CR2) was obtained from BRIT, DAE, GOI. Two syringes were loaded with 1 ml each of solution containing ^51^Cr and freshly prepared ascorbic acid (Sisco, India) (mass concentration of ascorbic acid =2 mg/ml), which were given holding time of two hours to ensure complete reduction of chromium to its trivalent form [6]. Activities of the loaded syringes were determined in a calibrated nucleonix gamma ray spectrometer having sodium iodide doped with thallium [NaI (TI)] based well type scintillation detector coupled to a single channel analyzer. All the activities were counted for 30 s and further converted as cps for calculations. Approximately 3000 cps of ^51^Cr (III) in 1 ml were injected through the IV cannula inserted into the rabbits’ right ear marginal vein, followed by 0.5 ml saline, to flush the injected ^51^Cr (III) solution into circulation. The syringe used for injection of the dose was measured for any activity remaining. The actual dose that was in effect administered was derived by subtracting the left-over activity in the syringe. An electronic timer was pre-set for a minute before the experiment and switched on immediately after injection. After a minute, 1 ml whole blood was obtained through the IV cannula, inserted into the rabbits’ left ear central artery and its activity was determined for 360 s. BV was calculated using the formula,

$$\text{BV}\left(\text{ml}\right){\text{V}}_{2}=\frac{{\text{C}}_{1}{\text{V}}_{1}}{{\text{C}}_{2}}$$

Where, C_l_ = cps/ml injected

C_2_ = cps/ml in the diluted blood

V_l_ = volume of tracer injected

V_2_ = blood volume

After initial measurement of BV using ^51^Cr (III), a repeat measurement was done exactly after sixty minutes, using the same technique as discussed above. An electronic timer was pre-set for sixty minutes and switched on immediately after the initial measurement using ^51^Cr (III). Two minutes before the completion of sixty minutes, 1 ml whole blood was obtained by a syringe through the IV cannula inserted into the left ear for measuring the background activity in the blood. At 60 min, approximately 3150 cps of ^51^Cr (III) was injected through the same IV cannula inserted in the right ear marginal vein, followed by 0.5 ml of saline to flush the injected solution into circulation. One minute later, 1 ml blood sample was obtained after dilution for repeat estimation of BV. The background sample, syringe used for injection of ^51^Cr (III) and the blood sample obtained after dilution was counted by the well counter for 360 s. The counts obtained from the syringe used for injection were subtracted to find the actual, activity introduced into the rabbit. Similarly, the counts obtained from the background sample were subtracted from the blood sample obtained after dilution. BV was obtained from the equation as mentioned above. The results obtained were compared with the BV measured by the initial ^51^Cr (III) measurement.

### Statistical analysis

Initial and repeat measurement of BV using ^51^Cr(III) were analysed using paired *t*-test and by the method of Bland and Altman plot for assessing the agreement between two methods [7]. Results were expressed in mean ± SD and *P* value <0.05 was considered as statistically significant. SPSS 15.0 was used for the analysis.

## Method validation

Mean BV values of initial measurement was 194.14 ± 48.44 ml or 88.12 ± 16.90 ml/kg body weight. Repeated mean BV values measured after 1 h was 183.16 ± 54.68 ml or 83.39 ± 22.80 ml/kg body weight. The average difference between the initial and repeated measurements was 10.93 ml (95% CI −3.33, 25.19 ml), which is not statistically significant (P = 0.128).

**Fig. 1:**
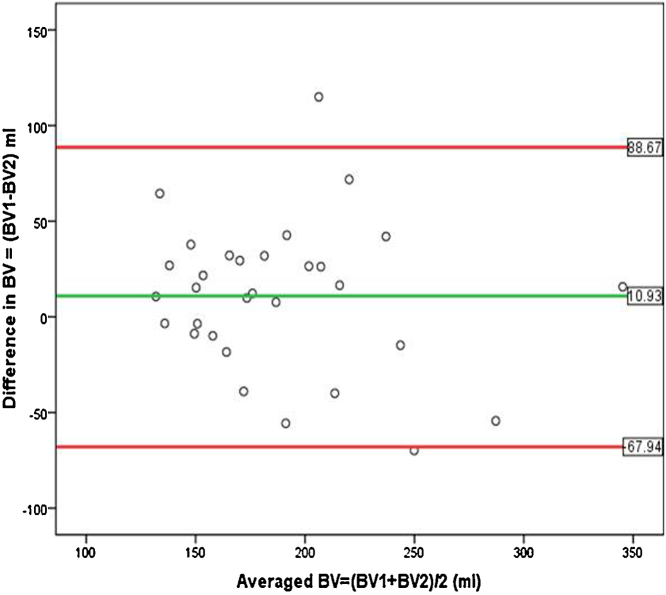
Bland and Altman plot: Repeatability of ^51^Cr (III) method (n = 31).

Bland and Altman plot (Fig. 1) given below shows that the limits of agreement were from −67.94 to 88.67 ml. For certain rabbits, the second reading were observed to be more than the first reading and this difference can go to a maximum of approximately 68 ml while for others the second reading was less than the first reading and the difference can go to a maximum of approximately 89 ml.

Rabbits were observed for any change in general appearance and behavioural patterns for four hours, from the initial administration of ^51^Cr(III). No changes were observed.

## Conclusion

BV measurements using low dose ^51^Cr (III) in rabbit model was found to be repeatable and reliable.
